# Supplementary material for: Identification and Functional Characterization of a Putative Alternative Oxidase (Aox) in Sporisorium reilianum f. sp. zeae
Source: J Fungi (Basel). 2022 Jan 31;8(2):148. doi: 10.3390/jof8020148 (PMC8877474; doi:10.3390/jof8020148)
Supplement: Supplementary file 1 [file jof-08-00148-s001.zip › jof-1561162-suppl-2.8.pdf]

Identification and functional characterization of a putative alternative oxidase (AOX) in *Sporisorium reilianum f. sp. zeae*

Hector Mendoza [1], Caroline D. Culver [1], Emma A. Lamb [1], Luke A. Schroeder [1], Sunita Khanal [2] and Michael H. Perlin [1,*]

[1]Department of Biology, Program on Disease Evolution, University of Louisville, Louisville, KY, USA;
[2]Division of Cardiovascular Medicine, School of Medicine, University of Maryland, Baltimore, MD;
*Correspondence: michael.perlin@louisville.edu

Supporting Information

Figure S1 – Growth inhibition assay of complemented SRZΔaox strains

Figure S2 - Fold change differences of AOX expression in teliospores and haploid cells of SRZ in reference to SRZ2

Table S1 – Strains used and generated in this study
Table S2 – Primers used in this study

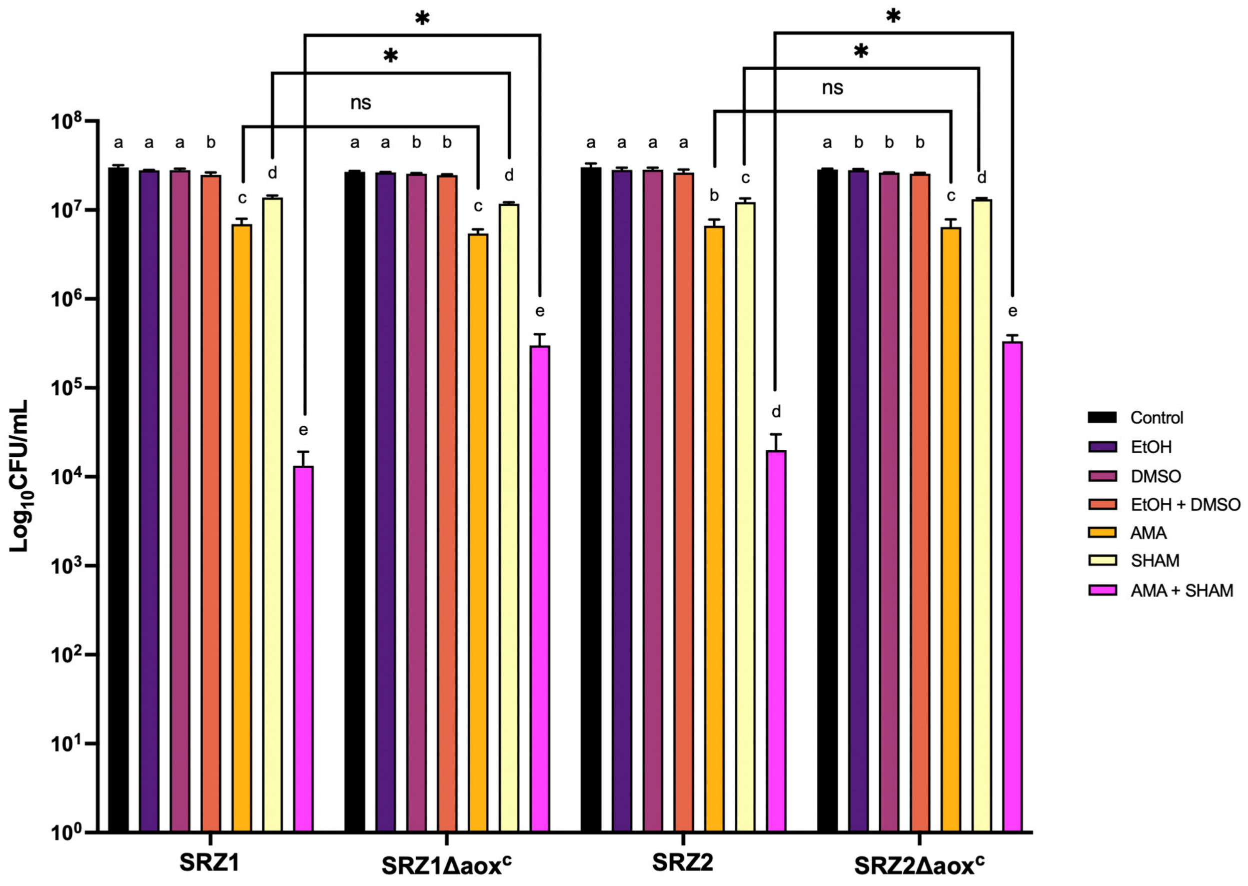

Figure S1: Growth inhibition assay of complemented SRZΔaox strains. 10^5 cells/mL were treated as indicated on the right and incubated at 28°C for 24 hours. Control groups consisted of untreated cells grown in PD broth. AMA and SHAM were used at concentrations of 50 μM and 2 mM, respectively. Cultures were then plated onto PD agar to determine the number of surviving colonies. Bars represent averages of biological triplicates with standard errors indicated. One-way ANOVA followed by Tukey's Multiple Comparison Test was performed in Graphpad 9.0. Letters above bars represent significant differences ($p < 0.05$) between the different treatments in reference to the corresponding control group of each strain. Comparisons of treatments between different strains are indicated by connecting black line brackets, with $p < 0.05$ represented by "*" (significant) and $p > 0.05$ represented by "ns" (not significant).

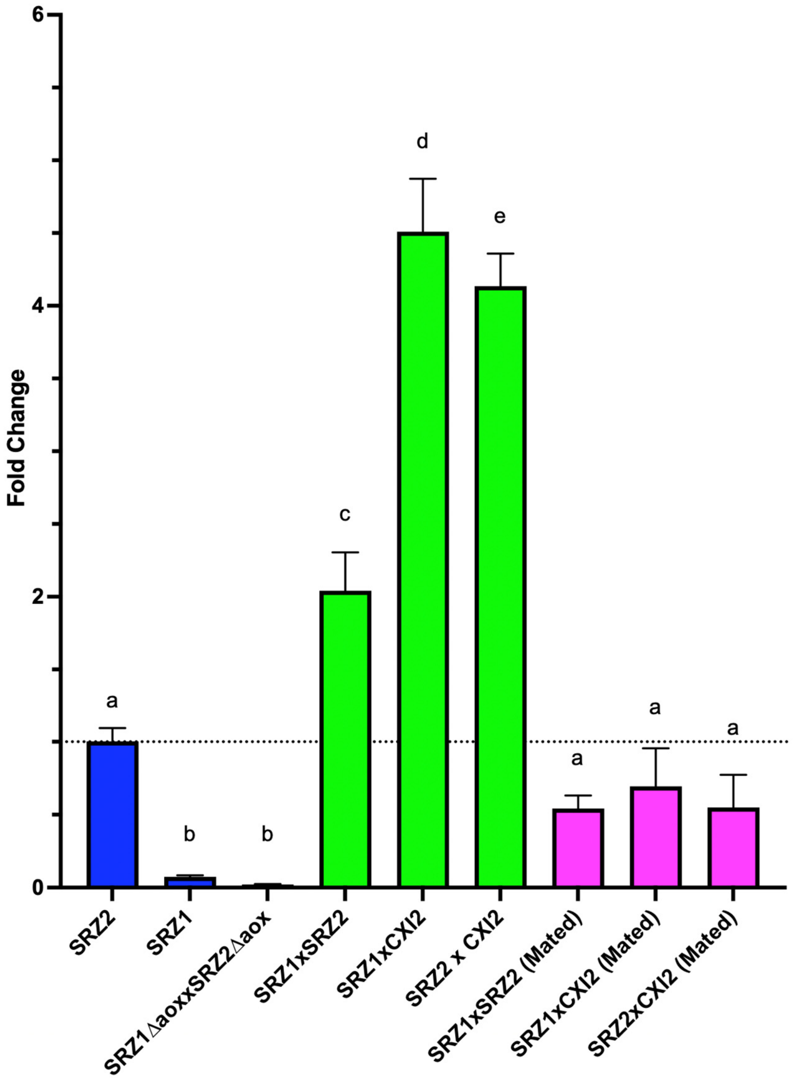

S2 – Fold change differences of *aox* expression in teliospores and haploid cells of SRZ in reference to SRZ2. Analysis was done relative to *gapdh* expression as endogenous control. Relative expression levels were calculated using the $2^{-\Delta\Delta Ct}$ method and were done in reference to strain SRZ2. Teliospores produced in a cross between *aox* deletion mutants and mated cells were included as controls. Colored bars correspond to cell type analyzed: blue = haploid cells, green = teliospores and pink = mated cells. One-way ANOVA followed by Tukey's Multiple Comparison Test was performed in Graphpad 9.0. Significant differences are indicated with letters above bars ($p < 0.05$).

Table S1 – SRZ strains used or generated in this study.

Strain	Genotype	Selectable marker	Source
SRZ2	a2b2	WT	[2]
SRZ1	a1b1	WT	[2]
SRZCXI2	a3b3	WT	Unpublished, from teliospores collected in China
SRZ2Δaox #1	a2b2Δaox::hph	Hygromycin	This study
SRZ1Δaox #2	a1b1Δaox::hph	Hygromycin	This study
SRZCXI2Δaox #4	a3b3Δaox::hph	Hygromycin	This study
SRZ2Δaoxc #1	a2b2Δaox::aoxsdh	Carboxin	This study
SRZ1Δaoxc #1	a2b2Δaox::aoxsdh	Carboxin	This study
SRZ2aox-eGFP #1	a2b2aox::hph	Hygromycin	This study
SRZ2aox-eGFP #1	a1b1aox::hpc	Hygromycin	This study

Table 2 – Primers used in this study.

Running #	Sequence (5′→3′)	Amplicon
oHM33	ACCATCCCTCTAAAACGACGGCCAGTGAAT	*hph* (2027 bp)
oHM34	AGTTCGATTCTGTGGAATTGTGAGCGGATA	
oHM35	CGTCGTTTTAGAGGGATGGTTGTGAAATGG	Upstream flanking region of *aox* (1285 bp)
oHM36	TTTTGATATCAAGCATGGTGACGAGGAGAT	
oHM37	CAATTCCACAGAATCGAACTGGCGAATGTC	Downstream flanking region of *aox* (1270 bp)
oHM38	TTCAATATTAATTAAAGGTGATGAAGGAACGAACG	
oHM39	CATGCTTGATATCAAAAGGCCGCGTTGCTG	*amp^r*, *ori* (1688 bp)
oHM40	ATCACCTTTAATTAATATTGAAAAAGGAAGAG	
oHM61	GAAAAGACCGTGGCTCTCC	*aox* for qRT-PCR
oHM62	GTGCTTCACTGGCATCGTC	
oHM70	TTTTGATATCCGACTTTTCGGGTGATTTTC	For AOX-eGFP fusion construct
oHM102	GAAGACCGCCATGGTGAGCAAGGGCGAG	
oHM103	CGTCGTTTTATTCTTGTGATTCGGGGACTC	
oHM104	TGCTCACCATGGCGGTCTTCTCAGCAGC	
oHM105	ATCACAAGAATAAAACGACGGCCAGTGAAT	
oHM146	CGAAAAGTCGGATATCAAAAGGCCGCGTTG	For AOX complementation construct
oHM147	TTTTGATATCCGACTTTTCGGGTGATTTTC	
oHM148	GCTCGATATTGGTGGTAAGGGTATCGGACA	
oHM149	CCTTACCACCAATATCGAGCACGTTGATGG	
oHM155	CCACAATCGTGAATCGAACTGGCGAATGTC	
oHM156	AGTTCGATTCACGATTGTGGCGAATCGCGG	
oHM30	TTCAATATTAATTAAGGTATGCCTCAGCTCAAAGG	
oYZ58	GGATTTCATCGGCAACTCAC	*gapdh* for qRT-PCR[1]
oYZ59	TACCACGAGACGAGCTTGAC	
